# Development of New Antiproliferative Compound against Human Tumor Cells from the Marine Microalgae *Nannochloropsis gaditana* by Applied Proteomics

**DOI:** 10.3390/ijms22010096

**Published:** 2020-12-24

**Authors:** Rafael Carrasco-Reinado, Almudena Escobar-Niño, Carlos Fajardo, Ines M. Morano, Francisco Amil-Ruiz, Gonzalo Martinez-Rodríguez, Carlos Fuentes-Almagro, Victoria Capilla, Lidia Tomás-Cobos, Laura Soriano-Romaní, Palmira Guarnizo, Roberto A. Vallejo, Francisco Javier Fernández-Acero

**Affiliations:** 1 Microbiology Laboratory, Institute of Viticulture and Agri-Food Research (IVAGRO), University of Cádiz, Pol. Río San Pedro s/n, 11510 Cádiz, Spain; rafael.carrasco@uca.es (R.C.-R.); almudena.escobar@uca.es (A.E.-N.); carfaqui07@yahoo.es (C.F.); inesm.morano@gmail.com (I.M.M.); 2 SCAI, University of Córdoba, Ramón y Cajal Building, Rabanales Campus, 14071 Córdoba, Spain; b72amruf@yahoo.es (F.A.-R.); b72fualc@uco.es (C.F.-A.); 3 Institute of Marine Science of Andalusia, Spanish Council for Scientific Research, ICMAN-CSIC, 11510 Cádiz, Spain; gonzalo.martinez@csic.es; 4 AINIA, Parque Tecnológico de Valencia, C.Benjamín Franklin 5-11, 46980 Paterna, Spain; vcapilla@ainia.es (V.C.); ltomas@ainia.es (L.T.-C.); lsoriano@ainia.es (L.S.-R.); 5 NeoALGAE Micro Seaweed Productos, Project Department, Planta I+D de Microalgas-UPT Litoral, Ctra. Faro Mesa Roldán s/n, 04140 Carboneras, Spain; guarnizogarciapalmira@gmail.com; 6 Research Project Manager of Innovation Department, Endesa Generación, S.A., Ribera del Loira 60, 28042 Madrid, Spain; roberto.andres@enel.com

**Keywords:** microalgae, tumor-antiproliferative, protein, biomedicine, applied proteomics, industrial application

## Abstract

Proteomics is a crucial tool for unravelling the molecular dynamics of essential biological processes, becoming a pivotal technique for basic and applied research. Diverse bioinformatic tools are required to manage and explore the huge amount of information obtained from a single proteomics experiment. Thus, functional annotation and protein–protein interactions are evaluated in depth leading to the biological conclusions that best fit the proteomic response in the system under study. To gain insight into potential applications of the identified proteins, a novel approach named “Applied Proteomics” has been developed by comparing the obtained protein information with the existing patents database. The development of massive sequencing technology and mass spectrometry (MS/MS) improvements has allowed the application of proteomics nonmodel microorganisms, which have been deeply described as a novel source of metabolites. Between them, *Nannochloropsis gaditana* has been pointed out as an alternative source of biomolecules. Recently, our research group has reported the first complete proteome analysis of this microalga, which was analysed using the applied proteomics concept with the identification of 488 proteins with potential industrial applications. To validate our approach, we selected the UCA01 protein from the prohibitin family. The recombinant version of this protein showed antiproliferative activity against two tumor cell lines, Caco2 (colon adenocarcinoma) and HepG-2 (hepatocellular carcinoma), proving that proteome data have been transformed into relevant biotechnological information. From *Nannochloropsis gaditana* has been developed a new tool against cancer—the protein named UCA01. This protein has selective effects inhibiting the growth of tumor cells, but does not show any effect on control cells. This approach describes the first practical approach to transform proteome information in a potential industrial application, named “applied proteomics”. It is based on a novel bioalgorithm, which is able to identify proteins with potential industrial applications. From hundreds of proteins described in the proteome of *N. gaditana*, the bioalgorithm identified over 400 proteins with potential uses; one of them was selected as UCA01, “in vitro” and its potential was demonstrated against cancer. This approach has great potential, but the applications are potentially numerous and undefined.

## 1. Introduction

Proteomics has become a crucial tool to reveal the molecular dynamics involved in a wide range of biological processes. The importance of proteomics in the understanding of biological processes is based on the functional information provided, which represents the overall protein content of an organism, including post-translational modifications, subcellular localization and protein–protein interactions, at a particular time and under a particular set of conditions [1,2], becoming a useful technique for early pathogenic bacterial identification, development of new antiviral drugs, identification of post-translational modifications, determination of the most abundant proteins of the host and pathogen during infection, finding of new therapeutic targets and biomarkers for the diagnosis of several illness, etc. [3,4,5,6].

On the other hand, nonmodel organisms have been increasingly described as interesting sources of novel bioproducts for human health, industry, agricultural applications and biotechnology. The rapid development of sequencing technology and improvements in the proteomics platforms have enabled the direct study of nonmodel microorganisms for the discovery of new specialized metabolites [7,8,9,10]. Among them, marine microalgae have recently awakened special interest due to their biological diversity, capacity to adapt their metabolism to a broad variety of environmental conditions, their unique metabolic pathways, as well as their high protein content. Moreover, they can potentially produce specific and new compounds, such as specific fatty acids, steroids, carotenoids, polysaccharides and specific proteins and peptides among others with biological activity (e.g., antiproliferative, cytotoxic, anticancer, photoprotective, anti-infective, etc.) [11]. Due to the potential applications of microalgae and being an eco-friendly product for the environment owing to their capacity to fix atmospheric CO_2_, microalgae have been positioned in the center of EU research policies and programmers, with the development of two main initiatives—Blue growth and Bio-Based Industries (BBIs). Blue growth is a long-term strategy to support sustainable growth in the marine and maritime sectors. BBI is a public-private partnership between the EU and the Bio-based Industries Consortium with the main aim of promoting initiatives to develop new bio-based products and markets. Both initiatives include the development of new products from microalgae biomass.

The potential of microalgae in the production of proteins with biotechnological application has been widely validated [12,13,14,15,16]. Nevertheless, there are only a few proteomics approaches to microalgae, most of them related to biofuels production [11,17,18,19,20] without any reference to the potential biotechnological use of this information [21,22,23,24,25,26,27,28,29,30,31,32,33,34,35,36]. Only 17 species have been studied from the several million different species of algae and microalgae [37]. The nonmodel organism used in this report was *Nannochloropsis gaditana*, that has been described as a producer of several high value compounds and has been pointed out as an alternative source of biomolecules for different biotechnological applications [38,39,40,41,42]. In addition, the availability of an *N. gaditana* genome sequence and transformation methods make the investigations into this microalga easier, such as for evaluating the performance of proteomic approaches [43]. However, there are only three previous proteomics analyses developed on *N. gaditana*, which have been mainly subproteomes of specific organelles, such as thylakoids [44]. Recently, our research group has reported the first complete proteome analysis of *N. gaditana* under industrial conditions [21].

Many computational techniques, databases and tools have been developed in order to resolve the growing size and complexity of the experimental proteomics datasets. Computational tools used to analyze proteomes are diverse, including fully automated and intuitive software tools that provide both quality control and biological interpretation of protein and Post-translational modification (PTM) level data at the same time [45]. However, tools for the optimal functional interpretation of proteomes in relation to several research questions are still scarce, making the development of new approaches necessary to increase the existing knowledge [46,47] and hopefully to develop biotechnological tools.

In the present work, the potential of microalgae has been combined with a new concept of using proteomics dataset, generating the “Applied proteomics” concept. Thus, proteomics data are combined with patents databases by a novel developed bioalgorithm, allowing the comparison of the identified proteins within a proteomics study, with those sequences registered in any patent document, revealing native proteins with direct industrial applications. To validate our approach, from the proteome of *N. gaditana*, the UCA01 protein from the prohibitin family was randomly selected by the algorithm. The recombinant protein UCA01 (patent number: 201930775) showed its antiproliferative activity against two tumor cell lines, Caco2 (colon adenocarcinoma) and HepG-2 (hepatocellular carcinoma), but not against nontumor cells, proving that proteome data can be transformed into relevant biotechnological information.

## 2. Results

### 2.1. Identified Protein Analysis

In a previous proteomic approach of *N. gaditana* performed by our group, 1950 proteins were identified, 655 of which were detected in all the employed samples and replicates [21]. In the present manuscript, this proteome was used to go through a second bioinformatic algorithm, where the search of identified proteins from *N. gaditana* was carried out against patented proteins database (Patent Protein database NRPL2 [48]). This new analysis showed that 488 proteins from the *N. gaditana* proteome showed sequence similarity with 14,718 proteins published in the Patent Protein NRL2 database (Supplementary Figure S1, sequence alignment of the sequencing results). Among those patents’ hits, 181 were related to “Cancer”; 122 were related to “Tumor”; 11 were related to “Metastases/Metastatic”. One of the *N. gaditana* proteins that showed similarity with some hits related to “Cancer” and “Tumor” was the protein annotated as “Prohibitin” (Uniprot accession number: W7TLA3).

Taking this into consideration, an analysis of the prohibitin sequences contained in *N. gaditana* genomes published in the databases was performed [49]. There were three nucleotide sequences of *N. gaditana* recorded in The European Nucleotide Archive (ENA) (https://www.ebi.ac.uk/ena), whose protein products belong to Prohibitin family of the stomatin, prohibitin, flotillin, and HflK/C (SPFH) superfamily. One of these coding sequences (CDSs) was EWM24278, the nucleotide sequence of the prohibitin presented in our algorithm search (uniprot accession: W7TLA3). This nucleotide sequence was the result of the *Nannochloropsis gaditana* strain:B-31 Genome sequencing (study: PRJNA170989) [50]. The others sequences, EWM26807 and EKU22077, were described by the *Nannochloropsis gaditana* strain:B-31 Genome sequencing (Study: PRJNA170989) [50] and the *Nannochloropsis gaditana* CCMP526 Genome sequencing (Study: PRJNA73791) [51], respectively. These last two sequences were the same, only differing in an initial 108 bp/36 aminoacid fragment that EWM26807 possesses compared to EKU22077 (Supplementary Figure S2. Comparative sequence between EWM26807 (W7TL69) and EKU22077 (K8YWQ7)).

**Figure 1:**
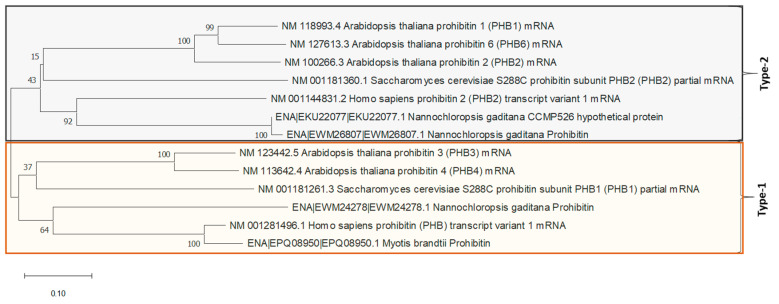
Evolutionary relationships of several described prohibitin nucleotides sequences and the 3 putative prohibitin of N. gaditana. Evolutionary analyses were conducted in MEGA X using pairwise deletion option 57. The phylogenetic tree was constructed using the Neighbor-Joining method [52] for the evolutionary history and the Maximum Composite Likelihood method [53] to compute the evolutionary distances. The bootstrap test was evaluated by 1000 replicates and the results are shown next to the branches [54].

Additionally, a phylogenetic analysis of the three sequences was performed in order to reveal which type of prohibitin each of them was (Figure 1). This analysis showed that EWM24278 clustered together with Prohibitin (PHB) type 1 (PHB1) genes and EWM26807 and EKU22077 clustered together with the type 2 (PHB2).

### 2.2. Heterologous Expression and Purification of Recombinant UCA01

Heterologous expression of UCA01 from cDNA (Supplementary Figure S3. Amplification of UCA01 DNA) was performed in order to purify and analyze its biological activity and the correct induction of the expression of *N. gaditana* recombinant protein by *E. coli* was checked by visualizing proteins in an acrylamide SDS-PAGE gel (Supplementary Figure S4. Expression of UCA01 in Rosetta gami), where it the induction of the recombinant prohibitin expression with the addition of a minimum concentration of 0.6 mM Isopropyl-*β*-d-1-thiogalactopyranoside (IPTG) was finally confirmed (Supplementary Figure S4. Expression of UCA01 in Rosetta gami). Bands E and F from SDS-PAGE gel (Supplementary Figure S5. UCA01 Purification) were extracted and analyzed by mass spectrometry (MS). This analysis showed the identification of peptides belonging to two different proteins.

**Figure 2:**
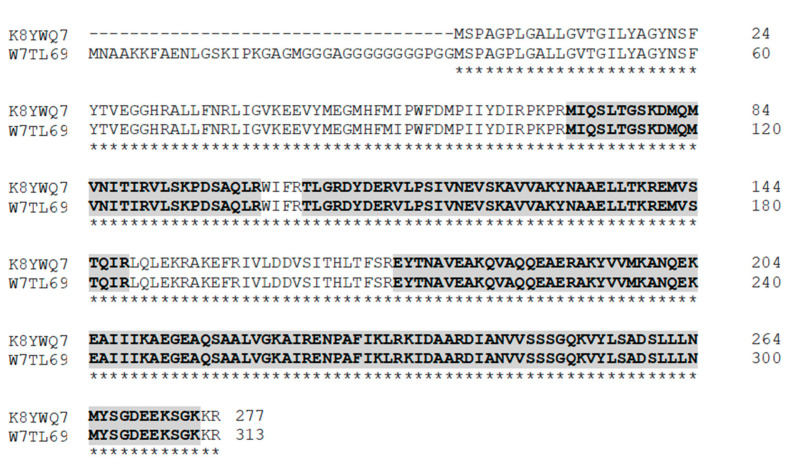
Coverage of K8YWQ7 (UCA01) and W7TL69. The coverage of K8YWQ7 and W7TL69 was performed using the peptides related to the N. gaditana prohibitin identified in the mass spectrometry (MS) analysis of the prohibitin purification SDS-PAGE gel.

One of them (Prohibitin OS = Nannochloropsis gaditana GN = Naga_100018g43 PE = 4 SV = 1/UniProt Accession: W7TL69) presented a 55.6% (28–29 peptides) coverage compared to a 3.7% (1 peptide) coverage of the other identified protein (elongation factor Tu, chloroplastic, OS = *Nannochloropsis gaditana*, GN = tufa, PE = 3, SV = 1/UniProt Accession: K9ZWB2), confirming the predominant presence of a type-2 prohibitin of *N. gaditana* in the purified elution sample. Comparing the amino acid sequence of two type-2 prohibitin proteins of *N. gaditana* (Uniprot Accession: W7TL69 and K8YWQ7), it was shown that the percentage of coverage of K8YWQ7 (UCA01) with the identified peptides related to prohibitin in the MS analysis was 62.82%, greater than the coverage of W7TL69 (Figure 2).

### 2.3. Antiproliferative Activity against Human Tumor Cells of N. gaditana Recombinant UCA01

**Figure 3:**
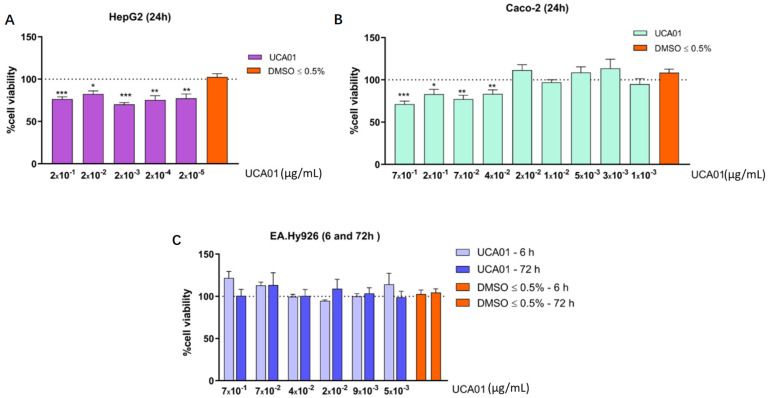
Antiproliferative effect of the recombinant UCA01 of *N. gaditana*. Antiproliferative effect was evaluated using different prohibitin or dimethylsulfoxide (DMSO) (vehicle) concentrations on human cell lines: (**A**) HepG2 (tumor cell line) after 24 h of contact; (**B**) Caco-2 (tumor cell line) after 24 h of contact; (**C**) EA.hy926 (nontumor/control cell line) after 6 h and 72 h of contact. The dashed line represents 100% cell viability obtained with each cell line without treatment. Data are expressed as means ± standard errors of the mean (SEM). For statistical analysis, Student’s *t*-test was used to compare each datum with the vehicle control DMSO (* *p* ≤ 0.05; ** *p* ≤ 0.01 and *** *p* ≤ 0.001).

Antiproliferative activity evaluation of the selected *N. gaditana* UCA01 was carried out on three different human cell lines: two tumor cell lines, Caco-2 (colon adenocarcinoma) and HepG2 (hepatocellular carcinoma) and a nontumor cell line (control cell line), EA.hy926 (endothelial cell line). The study was performed in each cell line under culture conditions indicated by the American type culture collection (ATCC, Manassas, VA, USA). Then, cells were incubated in 96-well plates at 37 °C, using different UCA01 protein concentrations and incubation times, as described in the materials and methods. After the incubation, cell viability was analyzed using a fluorescent detection method. This analysis showed that UCA01 inhibited the proliferation and cell viability of the human cancer cell lines, the liver cancer cell line HepG2 and the colon cancer cell line Caco-2 (Figure 3A,B), demonstrating for the first time the antiproliferative activity against human tumor cells of the *N. gaditana* recombinant protein UCA01 and of a type-2 PHB (PHB2). In addition, the antiproliferative effect in Caco-2 presented a trend of positive correlation with the concentration of recombinant UCA01—as protein concentration increases, so does the inhibition of proliferation in this cell line. On the other hand, *N. gaditana* UCA01 did not show antiproliferative effect against the EA.hy926 endothelial cell line—i.e., the nontumor cell line (Figure 3C). This result suggests that this protein has a selective effect, acting only on these tumor cell lines, but not against nontumor cells.

## 3. Discussion

Currently, proteomics has become a main tool for understanding the molecular dynamics involved in biological processes, in conjunction with other “omics” techniques [37,50,55]. The importance of proteomics to unravel biological processes is due to the functional information provided by the proteome, which shows the set of proteins involved at a particular time and under specific conditions, including post-translational modifications, subcellular location and protein–protein interactions [51,56].

On the other hand, microalgae have demonstrated the ability to help the main problems derived from human activities, such as the greenhouse effect or the protection of ecosystems [37]. These characteristics have turned microalgae into one of the key points of the “Blue growth” policies of the European Union—the revalorization of microalgae biomass [57]. The existence of several million species of microalgae, in comparison with terrestrial plants with 250,000 species, positions microalgae as ideal candidates to discover new products.

The assays carried out during this investigation present a new approach, adding to the possibilities of proteomics studies. To achieve this, the *N. gaditana* proteome was analyzed by means of a bioinformatics algorithm developed for this investigation. This algorithm allows the identification of existing proteins in *N. gaditana* with potential industrial applications, comparing the proteome with patent databases. This has led to the development of an important biotechnological tool, creating a new concept “Applied Proteomics”. This novel tool allows the transformation of proteome information into industrial information by highlining those listed proteins with potential biotechnological applications [21].

Once the proteins have been correlated with their potential industrial applications, it was necessary to conduct a study to select a protein of interest and test the hypothesis and the potential of “Applied Proteomics”. The protein UCA01 (K8YWQ7) was selected because it belongs to the prohibitin family, a group of proteins described in humans as multifunctional proteins. Among its functions, the upregulation of p53 protein activity, known as the guardian of the human genome, has been described. The p53 protein has the ability to control cell mitosis, reduce oxidative stress (antioxidant) and prevent mitochondrial dysfunction [58]. The protein UCA01 (K8YWQ7) has been compared with different prohibitins through different software tools. Clustal Omega software was used to compare UCA01 with other prohibitins presents in *N. gaditana* (Supplementary Figure S2. Comparative sequence between EWM26807(W7TL69) and EKU22077(K8YWQ7)), and MEGA X was used to compare with prohibitins present in other organisms (Figure 1). Results allowed us to catalog the protein UCA01 as prohibitin type number 2 (PHB2).

Prohibitin (PHB) is a protein that, in humans, is encoded by the *PHB* gene. The *PHB* gene has also been described in unicellular eukaryotes and pluricellular eukaryotes, such as animals, fungi or plants. Prohibitins are classified into two types based on their similarity to yeast PHB1 and PHB2, named Type-I and Type-II prohibitins, respectively [58,59]. Both PHB1 and PHB2 are members of a superfamily SPFH, which includes, in addition to prohibitins, stomatin, flotillin and HflKC. Each organism has at least one copy of each type of prohibitin gene [60,61]. Depending on the cellular localization, nucleus, cytosol, or mitochondria, PHB1 and PHB2 have distinctive functions, mainly within mitochondria. Due to their multiple functions in mitochondria, PHBs have been reported to be altered in various pathological conditions, such as cancer [62].

Several publications have showed differential expression of PHB1 and PHB2 in cancer cell lines compared to normal tissues, verifying that PHB1 and PHB2 are involved in biological processes of tumorigenesis, such as cancer cell proliferation [63]. PHB1 has been well identified as a potential tumor suppressor with antiproliferative activity in mammalian cells [64,65,66]. By contrast, PHB2 has only been highlighted as a promising antiproliferative agent, but this activity has not been demonstrated yet. This suggests that it could be interesting to perform a further analysis of the type-2 PHB gene. From the type-2 *N. gaditana* prohibitins sequences, EKU22077 was the only one contained in the gene database of NCBI. This is a searchable database of genes, focused on genomes that have been completely sequenced and that have an active research community to contribute gene-specific data. For this reason, EKU22077 (GenBank accession number: XM_005854224.1/GenPept accession number. XP_005854286/UniProt accession no. K8YWQ7) was the selected sequence to be deeply characterized by molecular and biochemical analysis, instead of selecting EWM26807 (GenPept accession number. EWM26807/UniProt accession: W7TL69). The selected sequence K8YWQ7 was renamed as UCA01.

The renamed protein UCA01 (patent number: 201930775) was synthesized in vitro and purified. Its biological activity was demonstrated against two human tumor lines (Caco-2 and HepG2), where the UCA01 protein was shown to inhibit the normal growth of both tumor lines (Figure 3). The effects of the UCA01 protein were also studied in a notumor human line, specifically the EA.hy926 line; in this cell line, the protein did not show any inhibition. So, this assay has demonstrated the selective character of UCA01, showing an antiproliferative effect on the Caco-2 and HepG2 tumor lines, while it showed no effect on the EA.hy926 nontumor line (Figure 3). Differences observed between Caco-2 and HepG2 in terms of effective antiproliferative concentrations are in line with recent previous reports showing differences in values obtained in these cell lines [67,68,69]. The relevance of discovering an antiproliferative protein in a microalga demonstrates the potential that microalgae have in the search for new compounds that could help humans and, of course, the potential of “Applied Proteomics” to identify them. The obtention of new compounds with antiproliferative activities against tumor cells from recombinant proteins opens a new path to replace existing treatments, which are very aggressive and with widely nondesirables side effects in patients [70]. In addition, this work describes, for the first time, the antiproliferative activities against human tumor cells of the *N. gaditana* recombinant protein belonging to the prohibitin family and of a type-2 PHB (PHB2) have been reported.

Therefore, the synthesis of a new tool (UCA01) to fight against tumor diseases, such as cancer, based on the new concept of applied proteomics, was achieved. The proteome of *N. gaditana*, has allowed us to identify 488 proteins with potential industrial applications.

## 4. Materials and Methods

### 4.1. Construction and Phylogenetics Analysis

In order to identify of *N. gaditana* proteins with industrial interest, a new algorithm was developed using the proteomic data previously generated by our group 14. The development of the algorithm was carried out at the Bioinformatics unit at University of Córdoba. This algorithm allows us to cross the proteins identified in Fernández-Acero et al.’s 2019 study with the United States patent database. This algorithm evaluates the putative industrial interest of the identified proteins by comparing sequence information with those proteins already involved in any patent document. Thus, a prohibitin coding protein of *N. gaditana* was selected according to its putative industrial application. The prohibitins presents in *N. gaditana* were searched in NCBI and Uniprot database (https://www.uniprot.org/). The existing sequences were compared using Clustal Omega (https://www.ebi.ac.uk/Tools/msa/clustalo/). 

For phylogenetic tree construction, multiple alignments were made with ClustalW and evolutionary analyses were conducted in MEGA X [71]. The evolutionary history was inferred using the Neighbor-Joining method [52]. The evolutionary distances were computed using the Maximum Composite Likelihood method [53] and branch support analysis was evaluated by 1000 ultrafast bootstrap replicates.

### 4.2. Heterologous Expression and Purification of Recombinant UCA01

#### 4.2.1. RNA Extraction, cDNA Synthesis and PCR Amplification

Total RNA was isolated following the indications of the NucleoSpin RNA^®^ commercial kit (Macherey-Nagel). The cDNA synthesis amplification was realized with the qScript^®^ commercial kit (Quanta Biosciences) through RT-PCR, following the kit instructions. PCR amplification was performed using the Phusion Flash High-Fidelity PCR Master Mix commercial kit (Thermo Scientific). The reaction conditions were: 2x Phusion Flash PCR Master Mix 25 μL, 10 μM, for Hypothetical protein (XM_005854224.1). “Forward primer”: 5′-GGC ATA TGT CTC CAG CAG GAC CGC TGG-3′ (cut site for NdeI) 2.5 μL, 10 μM “Reverse primer”: 5′-GGG TCG ACC TAC CGC TTC TTT CCA GAC TTC-3′ (cut site for SalI) 2.5 μL, cDNA (65 ng/μL) 2 μL and 18 μL water were used for a 50 μL reaction volume. The primers were designed with OligoCalc Software (version 3.27) based on the information of the prohibitin gene of *N. gaditana* (GenBank accession no. XM_005854224.1). The amplification conditions were: initial denaturation at 98 °C, 10 s, followed by 30 cycles at 98 °C, 1 s, 72 °C at 5 s and 72 °C at 15 s. Final extension cycle was at 72 °C for 3 min and the final storage step was at 4 °C. The amplified products were checked in agarose gel (1%), the electrophoresis have realized 1 h at 110 V, and stained with Gel-Red. Amplification fragments were sequenced (Stab Vida^®^, Lisboa Portugal).

Obtained sequences were checked using Blast (“BLAST: Basic Local Alignment Search Tool”).

#### 4.2.2. Amplified Fragment Purification

Purification was performed with GeneJET Gel Extraction Kit (Thermo Scientific, Waltham, MA, USA). The purified fragment from the gel bands was visualized by a 1% agarose gel run for 1 h at 110 V and stained with Gel-Red. 

#### 4.2.3. Digestion with Restriction Enzymes

In order to “linearize” the pET28a vector (Novagen, Sacramento, CA, USA) to allow for the ligation of the UCA01 fragment, the following reaction was carried out: 7 µL of pET28 (2 µg), 10 µL of 10X Buffer D (brand), 2 µL of NdeI (10 U/µL; brand), 2 µL of SalI (10 U/µL; brand) and 79 µL of water, for a total reaction volume of 100 µL. It was incubated for 4 h at 73 °C. Then, 11 µL of FastAp 10X buffer and 2 µL of alkaline phosphatase (1 U/µL; brand) were added and the reaction was incubated for 1 h at 37 °C. 

#### 4.2.4. Plasmid (pET28a) Plus Amplification Product Construction

Plasmid and amplification products were purified with the GeneJET Gel Extraction (Thermo Scientific) commercial kit. The products obtained after using the commercial kit were checked in agarose gel trough electrophoresis for 1 h at 110 V and the staining was performed with Gel-Red.

In total, 3 μL of vector (pET28a(+), 5369 bp) (12.8 ng/µL) with 10 μL of insert UCA01 (16.4 ng/µL) were mixed. The mix was incubated for 5 min at 70 °C, cooling afterwards on ice for 15 min. After, 5 μL of 5X Rapid Ligation Buffer, 1 μL ADN ligasa T4 enzyme (5 U/μL) (Thermo Scientific) and 13 μL nuclease free water were added. Total volume reaction was 25 μL and it was incubated at 22 °C for 1 h.

#### 4.2.5. Competent *E. coli* Cells Transformed with PET28a-UCA01 Construction

For the transformation procedure, competent *E. coli* top 10 (store strain, chemically competent, Invitrogen, USA) and *E. coli* Rosetta gami 1 (DE3) (expression strain, Novagen), cells were used, following the next protocol: 5 μL of the ligation mixture was added into 50 μL of the competent cells, gently mixed with the pipette and incubated on ice for 20 min. Then, a thermal shock of 42 °C was applied for exactly 45 s and then immediately incubated on ice for 5 min. Subsequently, 200 μL of LB medium were added, which was incubated for 1 h at 37 °C with stirring (200 rpm). Then, solid medium plates (LB/Kanamycin, 50 μg/mL) were spread with the mixture and incubated overnight at 37 °C.

#### 4.2.6. UCA01 Transform Isolation

To verify the correct transformation of the competent cells, the commercial kit GeneJET Miniprep (Thermo Scientific) was used to extract the isolated plasmid. Colony PCR were performed to verify that the transformation of both *E. coli* strains and the assembly of the plasmid with the insert had been correctly carried out.

#### 4.2.7. Induction of UCA01 Protein Expression

*E. coli* Rosseta gami 1 (DE3), transformed with the vector pET28Luci (complete gene) and with pET28, respectively, was incubated at 37 °C. Subsequently, fresh medium LB-kanamycin (50 μg/mL) was inoculated, and the culture was incubated at 37 °C with stirring (250 rpm) until reaching an optical density at 600 nm of 0.5–0.6. To induce protein expression, Isopropyl-*β*-d-1-thiogalactopyranoside (IPTG) was added until at a concentration of 1mM and incubated at 37 °C with stirring (250 rpm) for 2.5–3 h. Then, 1 mL of the induced culture was taken and mixed with 350 μL of resuspension buffer under native conditions (50 mM NaH2PO4-H2O, 300 mM NaCl, 10 mM imidazole, pH 8.0). The mix was frozen (−80 °C) and thawed four times, and then it was sonicated (60% amplitude) and centrifuged for 20 min at 8000 rpm. The soluble fraction was taken from the supernatant and the remaining pellet was resuspended in 350 μL of solubilization buffer under native conditions. The results were visualized by 10% SDS-PAGE, run at 200 V for 45 min and stained with Coomasie blue.

#### 4.2.8. UCA01 Recombinant Protein Purification

The purification of the recombinant protein was performed with His Spin Trap commercial kit (GE Healtcare), under denaturing conditions, and according to the manufacturer’s instruction. Binding buffer (20 mM Tris-HCl, 8 M urea, 500 mM NaCl, 5 mM imidazole, pH 8.0 + 1 mM β-mercaptoethanol) and elution buffer (20 mM Tris-HCl, 8 M urea, 500 mM NaCl, 500 mM imidazole, pH 8.0 + 1 mM β-mercaptoethanol) were used. The results were visualized by SDS-PAGE (10%), run at 200 V for 45 min and stained with Coomasie blue. Samples were then lyophilized for further testing. Six samples were purified with a content between 1.2 and 2.74 µg of protein (total = 10.46 µg of protein).Antiproliferative activity was exhibited against human tumor cells of *N. gaditana* recombinant UCA01.

Lyophilized samples were initially solubilized with dimethylsulfoxide (DMSO) at pH = 2, and subsequently, for application to cell cultures, diluted in DMEM culture medium (ATCC) supplemented with 100 U/mL penicillin and 100 µg/mL streptomycin (PAN Biotech), until a final DMSO concentration less than 0.5% was reached. Six samples were purified with a content of protein between 1.2 and 2.74 µg (total = 10.46 µg of protein).

The following cell lines obtained from the American type culture collection (ATCC, Manassas, VA, USA) were used:

- Human colorectal adenocarcinoma epithelial cell line: Caco-2 (ATCC^®^ HTB-5 37);
- Human hepatocellular carcinoma cell line: HepG2 (ATCC^®^-HB-8065);
- Human endothelial cell line EA.hy926 (ATCC^®^ CRL-2922 ™).

Cell lines were cultured at 37 °C and 5% CO_2_ in the cell culture medium recommended by the ATCC. For the Caco-2 cell line, the EMEM (Eagle’s Minimal Essential) medium was used and, for the HepG2 and for EA.hy926 cell lines, the DMEM (Dulbecco’s Modified Eagle) medium. These media were supplemented with 10% fetal bovine serum (FBS) and 100 U/mL penicillin and 100 µg/mL streptomycin.

The evaluation of the antiproliferative activity in the cells in contact with the UCA01 was analyzed in the indicated human cell lines. For this, the cells were grown in 96-well plates and incubated with a range between 0.7 and 0.003 µg/mL of UCA01 at different incubation times. In HepG2 and Caco-2 cells, the incubation time was 24 h, while in the nontumor cell line, EA.hy926, the incubation times were 6 and 72 h. After the incubation period, cell viability was evaluated by a fluori-colorimetric assay with the alamarBlue^®^ reagent (Invitrogen, Carlsbad, CA, USA). Fluorescence was measured at λexc/λem of 540/590 nm by a Fluostar Optima plate spectrofluorimeter (BMG Labtechnologies, Ortenberg, Germany). Given the direct relationship between fluorescence units and cell viability, the viability calculation was made with respect to the control cells without treatment, using the following formula:

% Cell Viability = (Sample Fluorescence Units/Control Fluorescence Units) × 100.

At least two independent experiments with triplicates were performed for each determination. Statistical significance between different conditions was assessed using the Student’s *t*-test with a Welch’s correction applied in case of significantly different variances (F test). A value of *p* ≤ 0.05 was considered significant.

## 5. Conclusions

Currently, proteomics is a fundamental tool for unraveling the molecular dynamics involved in different biological processes due to the functional information provided by the proteome [37,50,55]. This investigation has opened a new approach to the possibilities of proteomic studies. Analyzing the *N. gaditana* proteome with a new developed bioinformatics algorithm has allowed the identification of *N. gaditana* proteins with potential industrial applications. This has led to the development of a new concept “Applied Proteomics”, which has transformed the proteome of a microalga into an information of high industrial value. Analyzing the proteome of *N. gaditana* has permitted the identification of 488 proteins with potential industrial applications. Between them, the biological activity of the selected protein (UCA01) was demonstrated against two human tumor lines (caco-2 and HepG2), where the UCA01 protein showed antiproliferative activity against both tumor cell lines. This study has demonstrated, for the first time, the potential antitumor activity of the *N. gaditana* recombinant prohibitin UCA01 and the suggested antiproliferative activity of a type-2 PHB (PHB2). This represents the synthesis of a new first approach with great potential against tumor diseases, based on the new concept of applied proteomics.

## Data Availability

Used Proteomics data are available at ProteomeXchange dataset. ProteomeXchange title: Proteomics characterization of the non-model microorganism: Nannochloropsis gaditana. ProteomeXchange accession: PXD008499.

## Supplementary Materials

Supplementary materials are available online at https://www.mdpi.com/1422-0067/22/1/96/s1.
