# Supplementary material for: Development of New Antiproliferative Compound against Human Tumor Cells from the Marine Microalgae Nannochloropsis gaditana by Applied Proteomics
Source: Int J Mol Sci. 2020 Dec 24;22(1):96. doi: 10.3390/ijms22010096 (PMC7795124; doi:10.3390/ijms22010096)

# From proteome to application by applied proteomics. Validation through a new antitumor compound.

**Authors:**

**Rafael Carrasco-Reinado [1], Almudena Escobar-Niño [1], Carlos Fajardo [1]; Ines M. Morano [1], Francisco Amil-Ruiz [2], Gonzalo Martinez-Rodríguez [3], Carlos Fuentes-Almagro [2], Victoria Capilla [4], Lidia Tomás-Cobos [4], Laura Soriano-Romaní [4], Palmira Guarnizo [5], Roberto A. Vallejo [6] and Francisco Javier Fernández-Acero [1,*]**

**Supplementary Figure S1. Sequence alignment of the sequencing results.** CLUSTAL O(1.2.4) multiple sequence alignment of the sequencing results of the cloned prohibitin in pET28a. The sequencing results of the cloned prohibitin in pET28a were aligned with the nucleotide sequence XM_005854224.1 (GenPept: XP_005854286 /UniProt Accession: K8YWQ7) .

```
PLM00134506+T7              gtgccgcgcggcagccatatgtctccagcaggaccgctgggagccttgctaggtgtgacg    276
PLM00134505+T7              gtgccgcgcggcagccatatgtctccagcaggaccgctgggagccttgctaggtgtgacg    266
XM_005854224.1:35-868       ------------------ATGTCTCCAGCAGGACCGCTGGGAGCCTTGCTAGGTGTGACG    42
PLM00134507+T7              gtgccgcgcggcagccatatgtctccagcaggaccgctgggagccttgctaggtgtgacg    60
PLM00134509+T7              gtgccgcgcggcagccatatgtctccagcaggaccgctgggagccttgctaggtgtgacg    479
                                              ******************************************

PLM00134506+T7              ggcatcctttacgccgggtacaatagtttttacacggtggagggtgggcaccgagctctg    336
PLM00134505+T7              ggcatcctttacgccgggtacaatagtttttacacggtggagggtgggcaccgagctctg    326
XM_005854224.1:35-868       GGCATCCTTTACGCCGGGTACAATAGTTTTTACACGGTGGAGGGTGGGCACCGAGCTCTG    102
PLM00134507+T7              ggcatcctttacgccgggtacaatagtttttacacggtggagggtgggcaccgagctctg    120
PLM00134509+T7              ggcatcctttacgccgggtacaatagtttttacacggtggagggtgggcaccgagctctg    539
                            ************************************************************

PLM00134506+T7              ttgttcaacagattaatcggtgtgaaagaagaagtgtacatggagggaatgcattttatg    396
PLM00134505+T7              ttgttcaacagattaatcggtgtgaaagaagaagtgtacatggagggaatgcattttatg    386
XM_005854224.1:35-868       TTGTTCAACAGATTAATCGGTGTGAAAGAAGAAGTGTACATGGAGGGAATGCATTTTATG    162
PLM00134507+T7              ttgttcaacagattaatcggtgtgaaagaagaagtgtacatggagggaatgcattttatg    180
PLM00134509+T7              ttgttcaacagattaatcggtgtgaaagaagaagtgtacatggagggaatgcattttatg    599
                            ************************************************************

PLM00134506+T7              attccctggttcgacatgcccatcatttacgacatccgccccaagccccggatgatccag    456
PLM00134505+T7              attccctggttcgacatgcccatcatttacgacatccgccccaagccccggatgatccag    446
XM_005854224.1:35-868       ATTCCCTGGTTCGACATGCCCATCATTTACGACATCCGCCCCAAGCCCCGGATGATCCAG    222
PLM00134507+T7              attccctggttcgacatgcccatcatttacgacatccgccccaagccccggatgatccag    240
PLM00134509+T7              attccctggttcgacatgcccatcatttacgacatccgccccaagccccggatgatccag    659
                            ************************************************************

PLM00134506+T7              tccttgacaggaagcaaagacatgcaaatggtcaacatcaccatccgcgttttgtctaag    516
PLM00134505+T7              tccttgacaggaagcaaagacatgcaaatggtcaacatcaccatccgcgttttgtctaag    506
XM_005854224.1:35-868       TCCTTGACAGGAAGCAAAGACATGCAAATGGTCAACATCACCATCCGCGTTTTGTCTAAG    282
PLM00134507+T7              tccttgacaggaagcaaagacatgcaaatggtcaacatcaccatccgcgttttgtctaag    300
PLM00134509+T7              tccttgacaggaagcaaagacatgcaaatggtcaacatcaccatccgcgttttgtctaag    719
                            ************************************************************

PLM00134506+T7              cccgactcggctcaactccgctggatcttccgcaccttgggtcgcgactacgacgagcgt    576
PLM00134505+T7              cccgactcggctcaactccgctggatcttccgcaccttgggtcgcgactacgacgagcgt    566
XM_005854224.1:35-868       CCCGACTCGGCTCAACTCCGCTGGATCTTCCGCACCTTGGGTCGCGACTACGACGAGCGT    342
PLM00134507+T7              cccgactcggctcaactccgctggatcttccgcaccttgggtcgcgactacgacgagcgt    360
PLM00134509+T7              cccgactcggctcaactccgctggatcttccgcaccttgggtcgcgactacgacgagcgt    779
                            ************************************************************

PLM00134506+T7              gtcctcccttccatcgtcaacgaggtctccaaggccgtggtggccaagtacaacgccgcg    636
PLM00134505+T7              gtcctcccttccatcgtcaacgaggtctccaaggccgtggtggccaagtacaacgccgcg    626
XM_005854224.1:35-868       GTCCTCCCTTCCATCGTCAACGAGGTCTCCAAGGCCGTGGTGGCCAAGTACAACGCCGCG    402
PLM00134507+T7              gtcctcccttccatcgtcaacgaggtctccaaggccgtggtggccaagtacaacgccgcg    420
PLM00134509+T7              gtcctcccttccatcgtcaacgaggtctccaaggccgtggtggccaagtacaacgccgcg    839
                            ************************************************************

PLM00134506+T7              gagctcttgacgaagcgtgagatggtctccacccaaatccggttgcagttggagaagcgt    696
PLM00134505+T7              gagctcttgacgaagcgtgagatggtctccacccaaatccggttgcagttggagaagcgt    686
XM_005854224.1:35-868       GAGCTCTTGACGAAGCGTGAGATGGTCTCCACCCAAATCCGGTTGCAGTTGGAGAAGCGT    462
PLM00134507+T7              gagctcttgacgaagcgtgagatggtctccacccaaatccggttgcagttggagaagcgt    480
PLM00134509+T7              gagctcttgacgaagcgtgagatggtctccacccaaatccggttgcagttggagaagcgt    899
                            ************************************************************

PLM00134506+T7              gcgaaggagtttcggatcgtcctggacgacgtgtcgatcacccacttgaccttctcccgg    756
PLM00134505+T7              gcgaaggagtttcggatcgtcctggacgacgtgtcgatcacccacttgaccttctcccgg    746
XM_005854224.1:35-868       GCGAAGGAGTTTCGGATCGTCCTGGACGACGTGTCGATCACCCACTTGACCTTCTCCCGG    522
PLM00134507+T7              gcgaaggagtttcggatcgtcctggacgacgtgtcgatcacccacttgaccttctcccgg    540
PLM00134509+T7              gcgaaggagtttcggatcgtcctggacgacgtgtcgatcacccacttgaccttctcccgg    959
                            ************************************************************

PLM00134506+T7              gagtacacgaacgcggtcgaggccaagcaagttgctcaacaggaagccgagcgcgcgaaa    816
PLM00134505+T7              gagtacacgaacgcggtcgaggccaagcaagttgctcaacaggaagccgagcgcgcgaaa    806
XM_005854224.1:35-868       GAGTACACGAACGCGGTCGAGGCCAAGCAAGTTGCTCAACAGGAAGCCGAGCGCGCGAAA    582
PLM00134507+T7              gagtacacgaacgcggtcgaggccaagcaagttgctcaacaggaagccgagcgcgcgaaa    600
PLM00134509+T7              gagtacacgaacgcggtcgaggccaagcaagttgctcaacaggaagccgagcgcgcgaaa    1019
                            ************************************************************

PLM00134506+T7              tatgtggtaatgaaagcgaaccaagaaaaggaagccatcattatcaaagcggagggagag    876
PLM00134505+T7              tatgtggtaatgaaagcgaaccaagaaaaggaagccatcattatcaaagcggagggagag    866
XM_005854224.1:35-868       TATGTGGTAATGAAAGCGAACCAAGAAAAGGAAGCCATCATTATCAAAGCGGAGGGAGAG    642
PLM00134507+T7              tatgtggtaatgaaagcgaaccaagaaaaggaagccatcattatcaaagcggagggagag    660
PLM00134509+T7              tatgtggtaatgaaagcgaaccaagaaaaggaagccatcattatcaaagcggagggagag    1079
                            ************************************************************

PLM00134506+T7              gcccaatccgctgctctggtgggtaaggccattcgagagaatcctgcttttatcaagctg    936
PLM00134505+T7              gcccaatccgctgctctggtgggtaaggccattcgagagaatcctgcttttatcaagctg    926
XM_005854224.1:35-868       GCCCAATCCGCTGCTCTGGTGGGTAAGGCCATTCGAGAGAATCCTGCTTTTATCAAGCTG    702
PLM00134507+T7              gcccaatccgctgctctggtgggtaaggccattcgagagaatcctgcttttatcaagctg    720
PLM00134509+T7              gcccaatccgctgctctggtgggtaaggccattcgagagaatcctgcttttatcaagctg    1139
                            ************************************************************

PLM00134506+T7              cgcaaaatcgacgcagccagggacattgcgaatgtcgtgtcctcttcgggtcagaaggtc    996
PLM00134505+T7              cgcaaaatcgacgcagccagggacattgcgaatgtcgtgtcctcttcgggtcagaaggtc    986
XM_005854224.1:35-868       CGCAAAATCGACGCAGCCAGGGACATTGCGAATGTCGTGTCCTCTTCGGGTCAGAAGGTC    762
PLM00134507+T7              cgcaaaatcgacgcagccagggacattgcgaatgtcgtgtcctcttcgggtcagaaggtc    780
PLM00134509+T7              cgcaaaatcgacgcagccagggacattgcgaatgtcgtgtcctcttcgggtcagaaggtc    1199
                            ************************************************************

PLM00134506+T7              tatctgtctgcggactccctcttgttgaatatgtactcgggcgacgaagagaagtctgga    1056
PLM00134505+T7              tatctgtctgcggactccctcttgttgaatatgtactcgggcgacgaagagaagtctgga    1046
XM_005854224.1:35-868       TATCTGTCTGCGGACTCCCTCTTGTTGAATATGTACTCGGGCGACGAAGAGAAGTCTGGA    822
PLM00134507+T7              tatctgtctgcggactccctcttgttgaatatgtactcgggcgacgaagagaagtctgga    840
PLM00134509+T7              tatctgtctgcggactccctcttgttgaatatgtactcgggcgacgaagagaagtctgga    1259
                            ************************************************************

PLM00134506+T7              aagaagcggtaggtcgacaagcttgcggccgcactcgagcaccaccaccaccaccactga    1116
PLM00134505+T7              aagaagcggtaggtcgacaagcttgcggccgcactcgagcaccaccaccaccaccactga    1106
XM_005854224.1:35-868       AAGAAGCGGTAG------------------------------------------------    834
PLM00134507+T7              aagaagcggtaggtcgacaagcttgcggccgcactcgagcaccaccaccaccaccactga    900
PLM00134509+T7              aagaagcggtaggtcgacaagcttgcggccgcactcgagcaccaccaccaccaccactga    1319
                            ************
```

**Supplementary Figure S2. Comparative sequence between EWM26807(W7TL69) and EKU22077(K8YWQ7).** CLUSTAL O (1.2.4) multiple sequence alignment of EWM26807(W7TL69) and EKU22077(K8YWQ7) (A) nucleotides sequences; and (B) amino acid sequences.

**A.**

```
ENA|EKU22077|EKU22077.1       ------------------------------------------------------------    0
ENA|EWM26807|EWM26807.1       ATGAATGCAGCCAAGAAGTTTGCCGAGAACCTGGGTAGCAAAATTCCCAAGGGTGCTGGC    60

ENA|EKU22077|EKU22077.1       ------------------------------------------------ATGTCTCCAGCA    12
ENA|EWM26807|EWM26807.1       ATGGGGGGTGGCGCGGGTGGCGGGGGTGGAGGGGGTGGCCCCGGGGGCATGTCTCCAGCA    120
                                                                              ************

ENA|EKU22077|EKU22077.1       GGACCGCTGGGAGCCTTGCTAGGTGTGACGGGCATCCTTTACGCCGGGTACAATAGTTTT    72
ENA|EWM26807|EWM26807.1       GGACCGCTGGGAGCCTTGCTAGGTGTGACGGGCATCCTTTACGCCGGGTACAATAGTTTT    180
                              ************************************************************

ENA|EKU22077|EKU22077.1       TACACGGTGGAGGGTGGGCACCGAGCTCTGTTGTTCAACAGATTAATCGGTGTGAAAGAA    132
ENA|EWM26807|EWM26807.1       TACACGGTGGAGGGTGGGCACCGAGCTCTGTTGTTCAACAGATTAATCGGTGTGAAAGAA    240
                              ************************************************************

ENA|EKU22077|EKU22077.1       GAAGTGTACATGGAGGGAATGCATTTTATGATTCCCTGGTTCGACATGCCCATCATTTAC    192
ENA|EWM26807|EWM26807.1       GAAGTGTACATGGAGGGAATGCATTTTATGATTCCCTGGTTCGACATGCCCATCATTTAC    300
                              ************************************************************

ENA|EKU22077|EKU22077.1       GACATCCGCCCCAAGCCCCGGATGATCCAGTCCTTGACAGGAAGCAAAGACATGCAAATG    252
ENA|EWM26807|EWM26807.1       GACATCCGCCCCAAGCCCCGGATGATCCAGTCCTTGACAGGAAGCAAAGACATGCAAATG    360
                              ************************************************************

ENA|EKU22077|EKU22077.1       GTCAACATCACCATCCGCGTTTTGTCTAAGCCCGACTCGGCTCAACTCCGCTGGATCTTC    312
ENA|EWM26807|EWM26807.1       GTCAACATCACCATCCGCGTTTTGTCTAAGCCCGACTCGGCTCAACTCCGCTGGATCTTC    420
                              ************************************************************

ENA|EKU22077|EKU22077.1       CGCACCTTGGGTCGCGACTACGACGAGCGTGTCCTCCCTTCCATCGTCAACGAGGTCTCC    372
ENA|EWM26807|EWM26807.1       CGCACCTTGGGTCGCGACTACGACGAGCGTGTCCTCCCTTCCATCGTCAACGAGGTCTCC    480
                              ************************************************************

ENA|EKU22077|EKU22077.1       AAGGCCGTGGTGGCCAAGTACAACGCCGCGGAGCTCTTGACGAAGCGTGAGATGGTCTCC    432
ENA|EWM26807|EWM26807.1       AAGGCCGTGGTGGCCAAGTACAACGCCGCGGAGCTCTTGACGAAGCGTGAGATGGTCTCC    540
                              ************************************************************

ENA|EKU22077|EKU22077.1       ACCCAAATCCGGTTGCAGTTGGAGAAGCGTGCGAAGGAGTTTCGGATCGTCCTGGACGAC    492
ENA|EWM26807|EWM26807.1       ACCCAAATCCGGTTGCAGTTGGAGAAGCGTGCGAAGGAGTTTCGGATCGTCCTGGACGAC    600
                              ************************************************************

ENA|EKU22077|EKU22077.1       GTGTCGATCACCCACTTGACCTTCTCCCGGGAGTACACGAACGCGGTCGAGGCCAAGCAA    552
ENA|EWM26807|EWM26807.1       GTGTCGATCACCCACTTGACCTTCTCCCGGGAGTACACGAACGCGGTCGAGGCCAAGCAA    660
                              ************************************************************

ENA|EKU22077|EKU22077.1       GTTGCTCAACAGGAAGCCGAGCGCGCGAAATATGTGGTAATGAAAGCGAACCAAGAAAAG    612
ENA|EWM26807|EWM26807.1       GTTGCTCAACAGGAAGCCGAGCGCGCGAAATATGTGGTAATGAAAGCGAACCAAGAAAAG    720
                              ************************************************************

ENA|EKU22077|EKU22077.1       GAAGCCATCATTATCAAAGCGGAGGGAGAGGCCCAATCCGCTGCTCTGGTGGGTAAGGCC    672
ENA|EWM26807|EWM26807.1       GAAGCCATCATTATCAAAGCGGAGGGAGAGGCCCAATCCGCTGCTCTGGTGGGTAAGGCC    780
                              ************************************************************

ENA|EKU22077|EKU22077.1       ATTCGAGAGAATCCTGCTTTTATCAAGCTGCGCAAAATCGACGCAGCCAGGGACATTGCG    732
ENA|EWM26807|EWM26807.1       ATTCGAGAGAATCCTGCTTTTATCAAGCTGCGCAAAATCGACGCAGCCAGGGACATTGCG    840
                              ************************************************************

ENA|EKU22077|EKU22077.1       AATGTCGTGTCCTCTTCGGGTCAGAAGGTCTATCTGTCTGCGGACTCCCTCTTGTTGAAT    792
ENA|EWM26807|EWM26807.1       AATGTCGTGTCCTCTTCGGGTCAGAAGGTCTATCTGTCTGCGGACTCCCTCTTGTTGAAT    900
                              ************************************************************

ENA|EKU22077|EKU22077.1       ATGTACTCGGGCGACGAAGAGAAGTCTGGAAAGAAGCGGTAG    834
ENA|EWM26807|EWM26807.1       ATGTACTCGGGCGACGAAGAGAAGTCTGGAAAGAAGCGGTAG    942
                              ******************************************
```

**B.**

```
tr|K8YWQ7|K8YWQ7_NANGC        ------------------------------------MSPAGPLGALLGVTGILYAGYNSF    24
tr|W7TL69|W7TL69_9STRA        MNAAKKFAENLGSKIPKGAGMGGGAGGGGGGGPGGMSPAGPLGALLGVTGILYAGYNSF    60
                                                                  ************************

tr|K8YWQ7|K8YWQ7_NANGC        YTVEGGHRALLFNRLIGVKEEVYMEGMHFMIPWFDMPIIYDIRPKPRMIQSLTGSKDMQM    84
tr|W7TL69|W7TL69_9STRA        YTVEGGHRALLFNRLIGVKEEVYMEGMHFMIPWFDMPIIYDIRPKPRMIQSLTGSKDMQM    120
                              ************************************************************

tr|K8YWQ7|K8YWQ7_NANGC        VNITIRVLSKPDSAQLRWIFRTLGRDYDERVLPSIVNEVSKAVVAKYNAAELLTKREMVS    144
tr|W7TL69|W7TL69_9STRA        VNITIRVLSKPDSAQLRWIFRTLGRDYDERVLPSIVNEVSKAVVAKYNAAELLTKREMVS    180
                              ************************************************************

tr|K8YWQ7|K8YWQ7_NANGC        TQIRLQLEKRAKEFRIVLDDVSITHLTFSREYTNAVEAKQVAQQEAERAKYVVMKANQEK    204
tr|W7TL69|W7TL69_9STRA        TQIRLQLEKRAKEFRIVLDDVSITHLTFSREYTNAVEAKQVAQQEAERAKYVVMKANQEK    240
                              ************************************************************

tr|K8YWQ7|K8YWQ7_NANGC        EAIIIKAEGEAQSAALVGKAIRENPAFIKLRKIDAARDIANVVSSSGQKVYLSADSLLLN    264
tr|W7TL69|W7TL69_9STRA        EAIIIKAEGEAQSAALVGKAIRENPAFIKLRKIDAARDIANVVSSSGQKVYLSADSLLLN    300
                              ************************************************************

tr|K8YWQ7|K8YWQ7_NANGC        MYSGDEEKSGKKR  277
tr|W7TL69|W7TL69_9STRA        MYSGDEEKSGKKR  313
                              *************
```

**Supplementary Figure S3**. **Amplification of UCA01 DNA.** Fragment of Prohibitin (834 bp) amplified by RT-PCR from *N. gaditana* RNA. Lane M: Molecular weight ladder; Lane 1, 2 and 3: Prohibitin fragment (NCBI Accession: XM_005854224.1/UniProt Accession: K8YWQ7).

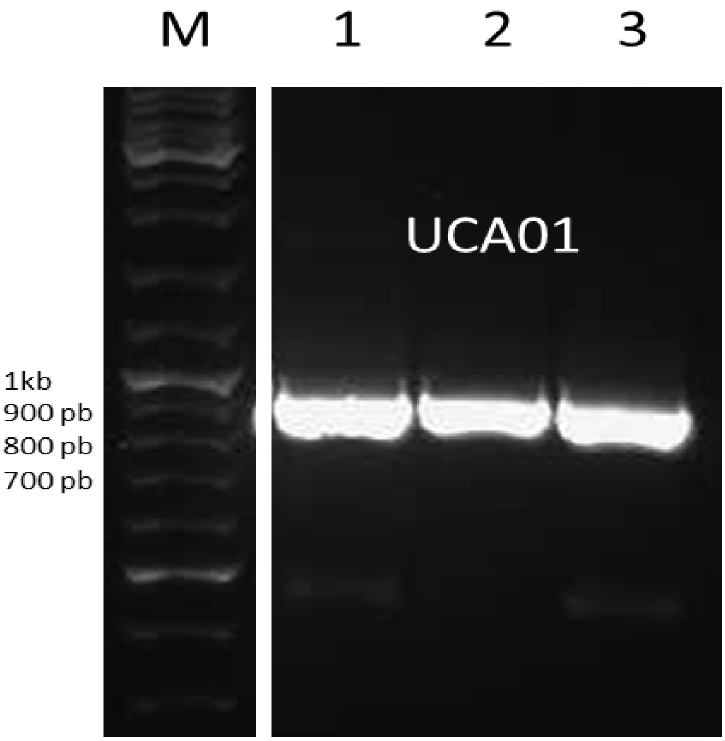


**Supplementary Figure S4**. **Expression of UCA01 in Rosetta gami**. 10% SDS-PAGE gel of *N. gaditana* UCA01 heterologous expression by *E. coli* Rosseta gami. Transformants of *E. coli* Rosseta gami with pET28a carrying *N. gaditana* prohibitin gene was cultured at 37ºC for 2.5-3 hours in LB with (Induction of plasmid expression) or without IPTG (No induction of plasmid expression). After mechanical lysis of the cultures, the soluble fraction was analysed by SDS-PAGE. Lane M: Molecular weight ladder. Lane 1: Induction of recombinant protein expression with 1mM of IPTG. Lane 2: No induction of recombinant protein expression (without IPTG). SDS-PAGE gel was run at 200V for 45 min and stained with Coomassie blue.

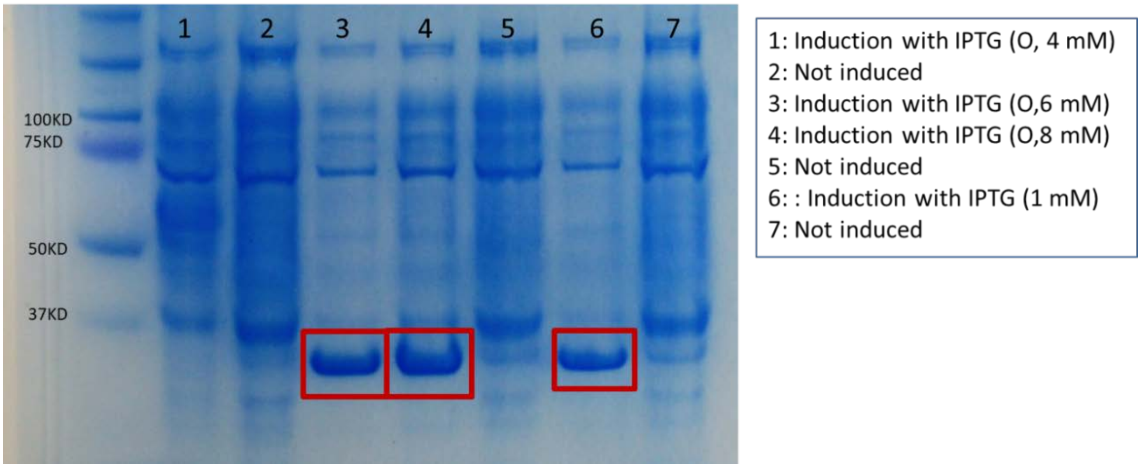

**Supplementary Figure S5**. **UCA01 Purification**. 10% SDS-PAGE gel of *N. gaditana* recombinant UCA01 purification. Induced culture *of E. coli* Rosseta gami expressing *N. gaditana* prohibitin gene was lysated and its soluble fraction was purified by metal ion affinity chromatography. The steps of the purification process were analysed by SDS-PAGE. Lane M: Molecular weight ladder. Lane A: No induced culture of transformant *E. coli* Rosseta gami (not purified fraction). Lane B: Induced culture of transformant *E. coli* Rosseta gami before purification. Lane C: load step of purification process using induced culture of transformant *E. coli* Rosseta gami. Lane D: Wash step of purification process using induced culture of transformant *E. coli* Rosseta gami. Lane E: First elution step of purification process using induced culture of transformant *E. coli* Rosseta gami. Lane F: Second elution step of purification process using induced culture of transformant *E. coli* Rosseta gami. SDS-PAGE gel was run at 200V for 45 min and stained with Coomassie blue.

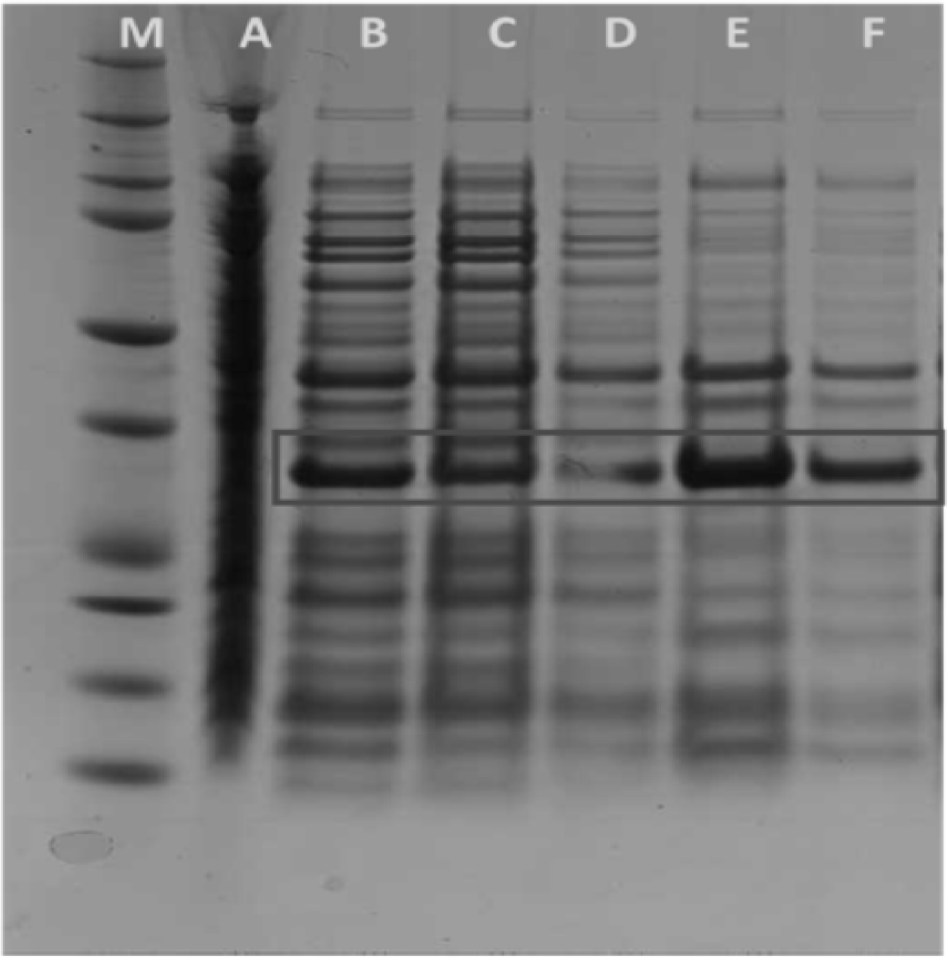

Supplement: Supplementary file 1 [file ijms-22-00096-s001.pdf]
